# Comparison of MCFA and Other Methods of Terminating Alcohol Fermentation and Their Influence on the Content of Carbonyl Compounds in Wine

**DOI:** 10.3390/molecules25235737

**Published:** 2020-12-04

**Authors:** Josef Licek, Mojmir Baron, Jiri Sochor

**Affiliations:** Department of Viticulture and Enology, Faculty of Horticulture, Mendel University in Brno, Valtická 337, 691 44 Lednice, Czech Republic; licek.josef@seznam.cz (J.L.); mojmir.baron@seznam.cz (M.B.)

**Keywords:** medium-chain fatty acids (MCFA), carbonyl compounds, alcohol fermentation

## Abstract

This study deals with the effects of the use of a mixture of medium-chain fatty acids (MCFA) at the end of the alcohol fermentation process on the content of carbonyl compounds in wine. During the experiment, the effects of the addition of MCFA at doses of 10 and 20 mg/L were compared to the termination of alcohol fermentation using cross-flow filtration and chilling treatments. Individual carbonyl compounds were determined by HPLC analysis. The experiment showed that the addition of MCFA caused a reduction of the acetaldehyde content compared to the chilling process, and a reduction of the diacetyl content compared to cross-flow filtration. Throughout the experiment, a lower level of total carbonyl compounds was observed after the addition of MCFA.

## 1. Introduction

In recent years, the wine industry has increasingly sought alternatives to sulfur dioxide to end alcohol fermentation. This is because sulfur dioxide is a major allergen that can cause health problems, and not only for allergy sufferers [1]. Medium-chain fatty acids (MCFA) could represent one such alternative for reducing sulfur dioxide doses.

MCFA are naturally occurring compounds in wine. Their inhibitory properties regarding yeasts and bacteria have been known for a long time [2,3], as has the path of their origin. They are produced by yeast activity as an intermediate in lipid metabolism [4,5]. They are mainly represented by hexanoic acid, octanoic acid, and decanoic acid (C6, C8, and C10, respectively). The presence of these fatty acids causes changes in the properties and functionality of cell membranes, making the yeast less resistant to stress factors, especially ethanol [6,7]. In addition, this effect increases with higher alcohol concentrations [8]. Therefore, the application of MCFA may represent a suitable supplement for ending alcohol fermentation. The positive effect of terminating alcohol fermentation through the addition of a mixture of C8, C10, and C12 has already been experimentally demonstrated [9].

However, the effect of MCFA on the carbonyl compound content has not yet been investigated. Carbonyl compounds in wine are mainly represented by acetaldehyde and 2-oxoacids (pyruvate and 2-oxoglutarate), as well as by acetoin and diacetyl. They mainly arise during alcohol fermentation by yeast metabolism. Acetaldehyde can also be formed by the oxidation of ethanol [10]. Additionally, high levels of carbonyl compounds are present in the juice and wines of botrytized grapes [11]. Due to the presence of the 2-oxoacids groups, carbonyl compounds are responsible for binding the free form of sulfur dioxide (HSO_3_^−^), rendering it ineffective. Therefore, a reduction of the carbonyl compound content could be key to reducing SO_2_ doses [12].

So far, only technological processes for reducing the content of carbonyl compounds (malolactic fermentation and sur lie) have been acknowledged [13]. Although some attempts to use phenylsulfonylhydrazine have been made, it is a substance that does not naturally occur in grapes or wine [14,15].

## 2. Results

### 2.1. Basic Parameters

Throughout the experiment, an analysis of the basic parameters in all variants was conducted. The results from the 1st and 9th day after the end of alcohol fermentation are shown in Table 1.

The table shows that the values of the basic parameters were not significantly different from variant to variant, with the exception of the residual sugar and tartaric acid content. The MCFA variants displayed a lower residual sugar content (11.0–11.5 g/L) than the chilling (13.0 g/L) and cross-flow filtration (14.8 g/L) variants. This was probably due to the mechanism of action of MCFA, as the inhibition of alcoholic fermentation occurred with a slight delay [7,9]. On the contrary, when yeasts were removed by cross-flow filtration, the effect was immediate. A lower tartaric acid content (2.5–2.6 g/L) was measured in the chilling variant, which was probably caused by the influence of the low temperature on the tartaric acid loss in the form of potassium hydrogen tartrate crystals [16].

### 2.2. Carbonyl Compounds

#### 2.2.1. Acetaldehyde

**Figure 1:**
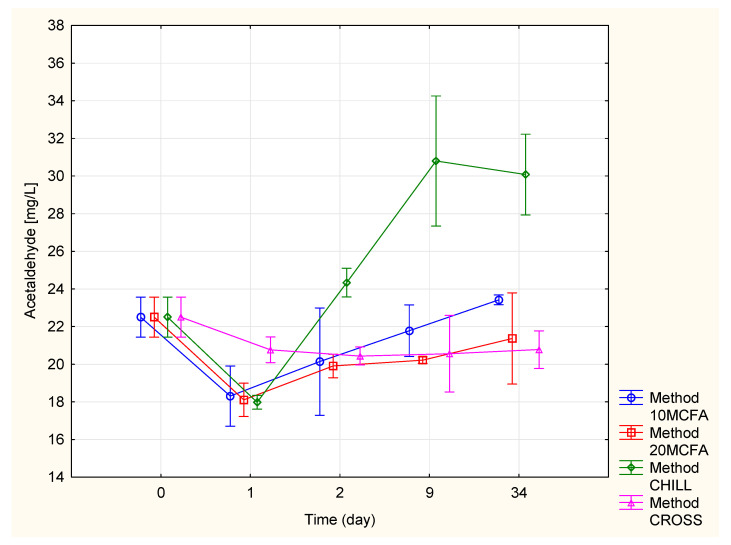
Changes in the acetaldehyde content.

The changes in the acetaldehyde content can be observed in Figure 1 and Table 2. On the 1st day, the highest concentration of acetaldehyde was measured in the cross-flow filtration variant (20.77 mg/L). However, over time, the content of the cross-flow filtration variant was stable, analogous to the 20 MCFA variant (21.37 mg/L). In contrast, the chilled variant exhibited an increase in the acetaldehyde content. On the 34th day, a considerably higher concentration (30.08 mg/L) was measured in the chilled variant (20.78 mg/L). This was caused by the yeasts that remained in the wine and slowly formed acetaldehyde to bind SO_2_ [12]. A significantly lower content was detected in the cross-flow (20.78 mg/L) and 20 MCFA (21.37 mg/L) variants. The reason for this was that the cross-flow filtration removed the yeasts from the wine. In the 20 MCFA variant, the yeasts were inhibited by the MCFA.

#### 2.2.2. Acetoin

**Figure 2:**
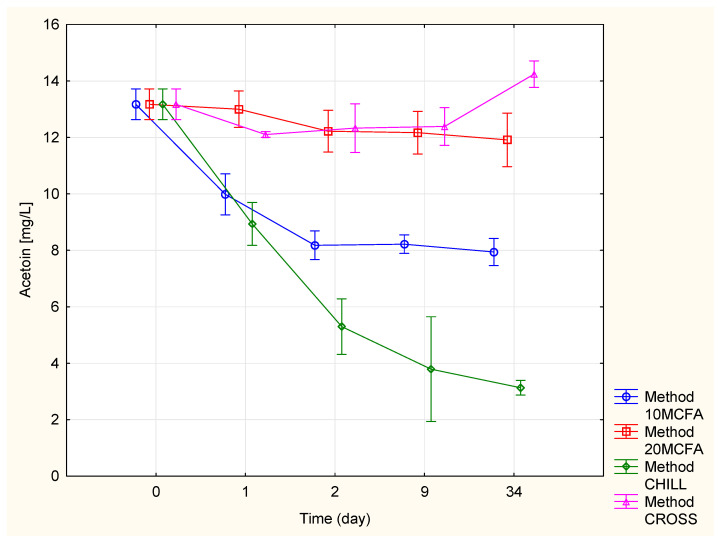
Changes in the acetoin content.

Figure 2 and Table 3 shows the changes in the acetoin content. The cross-flow filtration and 20 MCFA variants had relatively stable contents of acetoin (11.91–14.25 mg/L). The content of acetoin significantly decreased in the variant of 10 MCFA (up to 7.94 mg/L). The most noticeable decrease was measured in the chilled variant (3.14 mg/L). This trend was opposite to the trend seen for the acetaldehyde content. This may have been because the yeasts were producing NAD^+^ reduction coenzymes during acetaldehyde formation. In the next step, these coenzymes were involved in the reduction of acetoin and the production of butane-2,3-diol [17].

#### 2.2.3. Diacetyl

**Figure 3:**
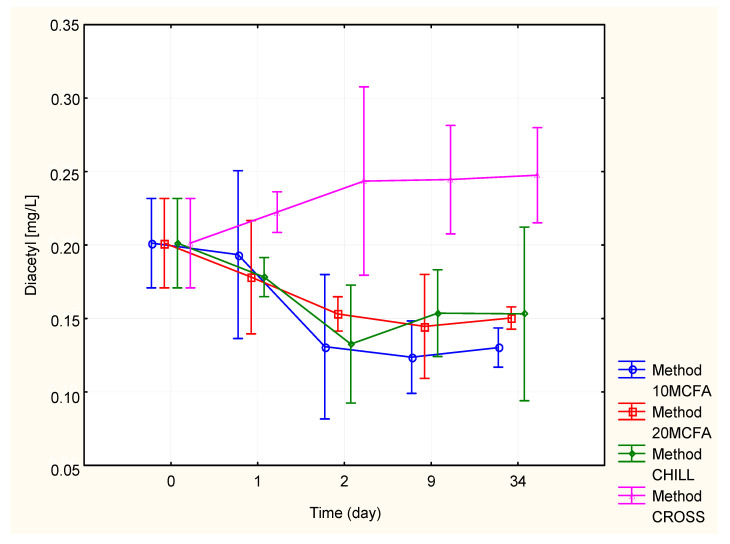
Changes in the diacetyl content.

The content of diacetyl is shown in Figure 3 and Table 4. The chilled, 10 MCFA, and 20 MCFA variants displayed a decrease of the diacetyl content (0.13–0.15 mg/L). The cross-flow filtration variant exhibited a significantly higher content of diacetyl (0.25 mg/L). This was probably due to the absence of yeast cells and reducing agents in the wine after cross-flow filtration, because the metabolism of yeasts or lees contact is responsible for a decrease in the diacetyl content [18].

#### 2.2.4. Total Carbonyl Compounds

**Figure 4:**
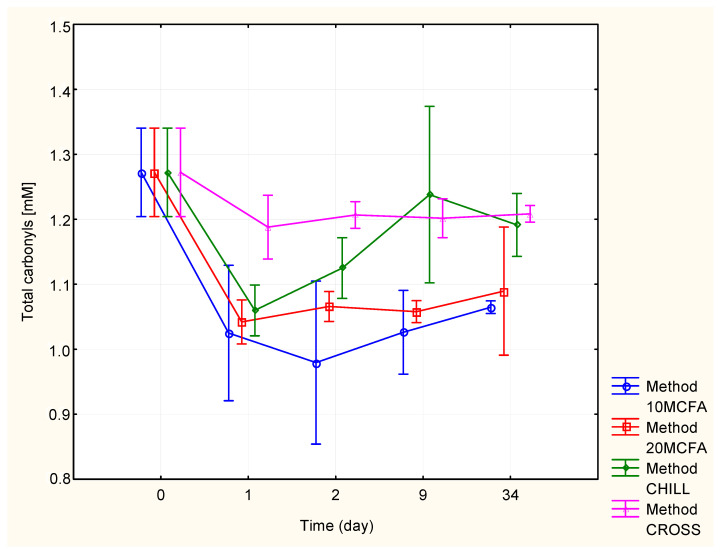
Changes in the total carbonyl content.

Figure 4 and Table 5 shows the content of total carbonyl compounds. On the 1st day, the highest concentration by a significant amount was found in the cross-flow filtration variant (1.18 mM/L). The development of the total carbonyl compound concentration was stable in this method. The other variants had significantly lower contents on the 1st day (1.03–1.06 mM/L). However, the chilled variant exhibited an increase in the later stages (1.24 mM/L). The lowest concentrations on the 9th and 34th day were measured in both MCFA variants (1.07 and 1.09 mM/L, respectively).

## 3. Materials and Methods

### 3.1. Design of the Experiment

The wines were produced in a winery called *Vinný dům Bzenec* (Bzenec, Czech Republic). The Rhein Riesling grapes were harvested in 2019. The must was clarified and inoculated by selected yeasts and the alcohol fermentation was controlled by cooling. The fermentation was stopped with the use of four different methods at a residual sugar content of approximately 15 g/L. During this phase, the control sample of the content of carbonyl compounds was taken. Next, the wine was distributed into four tanks, each with a volume of 25 hL. For each tank, a different method was applied, in order for a comparison to be carried out. The following methods were applied: The addition of 10 mg/L MCFA + 40 mg/L of SO_2_ (10 MCFA); the addition of 20 mg/L MCFA + 40 mg/L of SO_2_ (20 MCFA); chilling to 5 °C + 40 mg/L of SO_2_; and cross-flow filtration + 40 mg/L of SO_2_. After 24 h (1st day), the first samples were taken. Then, 100 g of SO_2_ gas (40 mg/L) from a pressure bottle was applied to each tank using a dispenser. Samples were then taken on the 2nd and the 9th day. After the 9th day, the content of free SO_2_ in each tank was adjusted to 35 mg/L. The final samples were taken on the 34th day.

### 3.2. Determination of the Carbonyl Compounds

The samples were processed in the laboratories of the Faculty of Horticulture in Lednice (Mendel University in Brno, Czech Republic). Individual carbonyl compounds were determined by HPLC Shimadzu LC-10A, and included acetaldehyde, pyruvate, 2-oxoglutaric acid, diacetyl, and acetoin. Their sum was expressed as the total carbonyl compound content.

The samples were adjusted by adding 100 µL of a 1 M NaOH solution to 100 µL of the wine, 100 µL of a 1.2 M H_2_SO_4_ solution, and 200 µL of 12 mM dinitrophenylhydrazine in acetonitrile in under 10 min. The mixture was allowed to temper at 60 °C for ten minutes. The treated samples were dosed in increments of 20 µL into the HPLC instrument. Samples were measured in triplicate.

Instrumentation: Shimadzu LC-10A Binary High-Pressure System; controller system: SCL-10Avp; two pumps: LC-10ADvp; column thermostat with a manual injection valve; Rheodyne: CTO-10ACvp; DAD detector: SPD-M10Avp; software: LCsolution; column: Alltech Alltima C183 µm.

### 3.3. Basic Parameters

The basic parameters (alcohol, residual sugar, titratable acids, pH, malic acid, tartaric acid, and lactic acid) were determined using the Alpha Bruker FTIR analyzer, which determines a substance’s content by spectrometry. One milliliter of the clear sample was used for this analysis. The application was performed using a syringe. The content of each substance was automatically evaluated by the software, depending on the calibration equations. All samples were measured in triplicate.

### 3.4. Preparation of the MCFA Mixture Solution

The mixture of MCFA was prepared according to European Patent No. 2681301. First, a mixture of fatty acids at the desired weight ratio (C8:C10:C12-2:7:1) was prepared. In this step, 100 g of a mixture of MCFA and 38 g of potassium hydroxide (KOH) were used per 1 L of the solution. The appropriate procedure was to weigh the MCFA mixture in a volumetric flask and gradually add the KOH solution, which had been dissolved in two thirds of the required water. After dissolution of the MCFA and temperature stabilization, the pH was optionally adjusted to a value above 10, and the diluted KOH solution was regulated to the desired volume. The resulting solution should have had an average density of 1.0128 kg/L [19].

## 4. Discussion

Acetaldehyde is the carbonyl compound that has the highest SO_2_-binding capacity; it constitutes up to 75% of all carbonyl compounds in white wine [10]. The dissociation constant (Kd) value is 2.4 × 10^−6^ mol/L. Other carbonyl compounds already in the wine have a value by the order of the magnitude lower-in pyruvate it is 0.3 × 10^−3^ mol/L and in 2-oxoglutarate it is 0.5 × 10^−3^ mol/L [20]. The acetaldehyde content values for the variants using MCFA ranged from 18.11 mg/L to 23.42 mg/L throughout the experiment. In comparison to the average values of 34 mg/L [21] and 33 mg/L [10], this represents a relatively significant reduction in acetaldehyde content.

Another positive effect of the MCFA was observed in the reduction of the acetoin and diacetyl content, which are responsible for the buttery tones in the aroma of wine. To some extent, they are formed during alcoholic fermentation [17], but much greater quantities are produced by lactic acid bacteria during malolactic fermentation [22].

In terms of the sensory activity, however, acetoin levels (3.14–14.25 mg/L) for all variants were well below the reported perception threshold of 150 mg/L [17], but the measured diacetyl content could be sensory-active. In the chilled and MCFA variants, the values were similar (0.13–0.15 mg/L), and were below the perception threshold reported by [23] for some white wines (0.18 mg/L). In contrast, the cross-flow filtration variant showed a higher content (0.25 mg/L). However, the perception threshold of diacetyl is strongly influenced by the style of wine [23]. Either way, the reduction of acetoin and diacetyl could be another positive effect of the addition of MCFA.

Malolactic fermentation is one of the most common and efficient technological operations for reducing the carbonyl compounds in wine. A study on the reduction of carbonyl compounds using this method showed an 87% reduction of the acetaldehyde content compared to the control variant, as well as a reduction in pyruvate and 2-oxoglutarate concentrations [13]. However, malolactic fermentation also increases the acetoin and diacetyl content [20]. This study demonstrated a 30% reduction in the acetaldehyde content using MCFA in comparison to the chilling process, which is a commonly used method for terminating alcohol fermentation [12]. Therefore, malolactic fermentation noticeably reduces the content of carbonyl compounds in wine. However, as it is a process that usually takes place after the end of alcoholic fermentation, it can hardly be applied as a support treatment to reduce the carbonyl compound content at the end of alcohol fermentation.

Furthermore, studies on reducing carbonyl compounds by extraction with phenylsulfonylhydrazine, which is extracted from lignin, have been published. A reduction in carbonyl compounds of up to 75% has been demonstrated; however, the experiment was carried out in laboratory conditions using already stabilized wine. Moreover, phenylsulfonylhydrazine is a compound which, unlike MCFA, does not naturally occur in wine [14,15].

None of the published studies are concerned with reducing the carbonyl compounds in connection with the end of alcohol fermentation. The obtained results demonstrate that the reduction of carbonyl compounds after using MCFA may be an incentive to further investigate the use of MCFA for assisting with alcohol fermentation termination and reducing the carbonyl compounds.

## 5. Conclusions

Based on the results, it can be said that the addition of MCFA to help stop the alcohol fermentation process led to a significant decrease in the carbonyl compound content in wine. After cross-flow filtration, the content of individual carbonyl compounds in wine was relatively stable. The chilling process led to a decrease in the content of acetaldehyde after an addition of SO_2_; however, after some days, the concentration increased. This was probably caused by the yeasts that remained in the wine and slowly formed acetaldehyde to bind the SO_2_. The development of the total carbonyl compound values was similar. After the adjustment of SO_2_, the values of the total carbonyls were comparable for the chilling and cross-flow filtration treatments. After cross-flow filtration, the wine did not contain any yeast cells that could reduce the carbonyl compounds, so their levels stayed high. In all sampling terms, the lowest value of total carbonyl compounds was measured when MCFA were applied.

## Figures and Tables

**Table 1:** Basic parameters of the wines.

Method	Day	Alcohol ^1^	Residual Sugar ^2^	Titratable Acids ^2^	pH	Malic Acid ^2^	Tartaric Acid ^2^	Lactic Acid ^2^
10 MCFA	1st	11.88	11.5	6.7	3.29	1.5	3.3	0.2
	9th	12.17	11.0	6.7	3.30	1.5	3.1	0.1
20 MCFA	1st	11.86	11.5	6.7	3.29	1.5	3.4	0.1
	9th	12.21	11.4	6.7	3.30	1.5	3.3	0.1
Chilling	1st	11.86	13.0	6.2	3.29	1.4	2.6	0.1
	9th	12.10	13.0	6.2	3.29	1.4	2.5	0.0
Cross-flow	1st	11.84	14.8	7.1	3.26	1.3	3.7	0.2
	9th	11.88	14.8	7.1	3.27	1.5	3.5	0.2

^1^ Content in % volume, and ^2^ content in g/L.

**Table 2:** Changes in acetaldehyde content. The average values (*n* = 3) were combined by contribution into homogeneous groups (a, b, c, and d), according to Fisher’s LSD (Least significant difference) test (α = 0.05).

Method	Control	1st Day	9th Day	34th Day
Acetaldehyde (mg/L) ± SD				
10 MCFA	22.50 ± 0.25 a	18.31 ± 0.37 a	21.78 ± 0.32 a	23.42 ± 0.06 b
20 MCFA	22.50 ± 0.25 a	18.11 ± 0.21 a	20.22 ± 0.04 a	21.37 ± 0.56 a
Chilling	22.50 ± 0.25 a	17.98 ± 0.09 a	30.80 ± 0.80 b	30.08 ± 0.50 c
Cross-flow	22.50 ± 0.25 a	20.77 ± 0.16 b	20.56 ± 0.47 a	20.78 ± 0.23 a

**Table 3:** Changes in acetoin content. The average values (*n* = 3) are supplemented by contribution into homogeneous groups (a, b, c, d) according to Fisher’s LSD (Least significant difference) test (α = 0.05).

Method	Control	1st Day	9th Day	34th Day
Acetoin (mg/L) ± SD				
10 MCFA	13.18 ± 0.13 a	9.98 ± 0.17 b	8.22 ± 0.08 b	7.94 ± 0.11 b
20 MCFA	13.18 ± 0.13 a	13.00 ± 0.15 d	12.17 ± 0.18 c	11.91 ± 0.22 c
Chilling	13.18 ± 0.13 a	8.94 ± 0.18 a	3.79 ± 0.43 a	3.13 ± 0.06 a
Cross-flow	13.18 ± 0.13 a	12.10 ± 0.02 c	12.39 ± 0.15 c	14.24 ± 0.11 d

**Table 4:** Changes in diacetyl content. The average values (*n* = 3) are supplemented by contribution into homogeneous groups (a, b, c, d) according to Fisher’s LSD (Least significant difference) test (α = 0.05).

Method	Control	1st Day	9th Day	34th Day
Diacetyl (mg/L) ± SD				
10 MCFA	0.20 ± 0.01 a	0.19 ± 0.09 a	0.12 ± 0.01 a	0.13 ± 0.00 a
20 MCFA	0.20 ± 0.01 a	0.18 ± 0.01 a	0.14 ± 0.01 a. b	0.15 ± 0.00 a
Chilling	0.20 ± 0.01 a	0.18 ± 0.00 a	0.15 ± 0.01 b	0.15 ± 0.01 a
Cross-flow	0.20 ± 0.01 a	0.22 ± 0.00 b	0.24 ± 0.01 c	0.25 ± 0.01 b

**Table 5:** Changes in total carbonyls content. The average values (*n* = 3) are supplemented by contribution into homogeneous groups (a, b, c, d) according to Fisher’s LSD (Least significant difference) test (α = 0.05).

Method	Control	1st Day	9th Day	34th Day
Total Carbonyls (mM/L) ± SD				
10 MCFA	1.27 ± 0.02 a	1.02 ± 0.02 a	1.03 ± 0.01 a	1.06 ± 0.00 a
20 MCFA	1.27 ± 0.02 a	1.04 ± 0.01 a	1.06 ± 0.00 a	1.09 ± 0.02 a
Chilling	1.27 ± 0.02 a	1.06 ± 0.01 a	1.24 ± 0.03 b	1.19 ± 0.01 b
Cross-flow	1.27 ± 0.02 a	1.19 ± 0.01 b	1.20 ± 0.01 b	1.21 ± 0.00 b
